# Anti-Müllerian Hormone Expression in Endometrial Cancer Tissue

**DOI:** 10.3390/ijms20061325

**Published:** 2019-03-15

**Authors:** Marek Gowkielewicz, Aleksandra Lipka, Aleksandra Piotrowska, Marta Szadurska-Noga, Jacek J. Nowakowski, Piotr Dzięgiel, Mariusz Krzysztof Majewski, Marcin Jozwik, Marta Majewska

**Affiliations:** 1 Department of Gynecology and Obstetrics, School of Medicine, Collegium Medicum, University of Warmia and Mazury in Olsztyn, 10-045 Olsztyn, Poland; aleksandra.lipka@uwm.edu.pl (A.L.); prof.jozwik@gmail.com (M.J.); 2 Division of Histology and Embryology, Department of Human Morphology and Embryology, Wroclaw Medical University, 50-368 Wroclaw, Poland; aleksandra.piotrowska@umed.wroc.pl (A.P.); piotr.dziegiel@umed.wroc.pl (P.D.); 3 Department of Pathomorphology, School of Medicine, Collegium Medicum, University of Warmia and Mazury in Olsztyn, 10-561 Olsztyn, Poland; szadurskamarta@gmail.com; 4 Department of Ecology & Environmental Protection, University of Warmia and Mazury in Olsztyn, 10–727 Olsztyn, Poland; jacek.nowakowski@uwm.edu.pl; 5 Department of Physiotherapy, Wroclaw University School of Physical Education, 51-612 Wroclaw, Poland; 6 Department of Human Physiology, School of Medicine, Collegium Medicum, University of Warmia and Mazury in Olsztyn, 10-082 Olsztyn, Poland; mariusz.majewski@uwm.edu.pl (M.K.M.); marta.majewska@uwm.edu.pl (M.M.)

**Keywords:** AMH, endometrial cancer, endometrium

## Abstract

Anti-Müllerian hormone (AMH) is a commonly known factor secreted by Sertoli cells, responsible for regression of the Müllerian ducts in male fetuses. AMH has also other functions in humans. In vivo and in vitro studies have shown that AMH inhibits cell cycle and induces apoptosis in cancers with AMH receptors. The aim of the study was to assess whether the tissue of pre-cancerous states of endometrium (PCS) and various histopathologic types of endometrial cancer (EC) exhibit the presence of AMH. We aimed to investigate whether the potential presence of the protein concerns menopausal women or those regularly menstruating, and whether is related to cancers with a good or a bad prognosis, as well as what other factors may influence AMH expression. The undertaken analysis was carried out on tissues retrieved from 232 women who underwent surgical treatment for PCS and EC. Tissues were prepared for immunohistochemical assessment with the use of a tissue microarrays method. AMH expression was confirmed in 23 patients with well differentiated endometrioid adenocarcinoma (G1), moderately differentiated endometrioid adenocarcinoma (G2), clear cell carcinoma (CCA) and nonatypical hyperplasia. AMH was not found in EC tissues in regularly menstruating women. An appropriately long mean period of breastfeeding in line with a prolonged period of hormonal activity had a positive effect on AMH expression. Our results may suggest that AMH is a factor which protects the organism against cancer, and should be further investigated as a potential prognosis marker and a therapeutic agent.

## 1. Introduction

Anti-Müllerian hormone (AMH) also known as Müllerian-Inhibiting Substance, is a well-studied regulatory molecule in reproductive functioning, especially in sexual differentiation during early embryonic development [1]. AMH is a 140 kDa dimeric glycoprotein encoded by a 2.75 kbp gene composed of five exons located within chromosome 19 p 13.3, which belongs to the transforming growth factor-beta (TGF-β) family [2,3,4,5]. To fulfil its biological role and to exert specific effect, AMH interacts with AMH type II specific (AMHRII) and AMH type I general (AMHRI) membrane receptors [6,7,8]. AMH is secreted by Sertoli cells of the male embryo testes as early as during the 8th–10th week of gestation [9,10,11]. This hormone is responsible for regression of the Müllerian ducts in the processes of apoptosis, auto-phagocytosis, cell migration and remodeling [12,13,14,15,16]. Postnatally, the serum levels of AMH increase significantly until puberty and then slowly decline throughout the rest of the man’s life [17]. In female embryos, absence of AMH allows the Müllerian ducts to develop into the uterus, the fallopian tubes, the upper third of the vagina and the outer lining of the ovaries [8,16]. Around the 36th week of gestation, granulosa cells of small growing ovarian follicles undergoing initial recruitment begin to secrete AMH [10,18,19]. Shortly after birth in females, the serum AMH concentration drops but it increases again around the age of two [18]. The serum AMH level reaches its peak at about the age of 25 years and as the woman approaches menopause a progressive decrease occurs, and the substance ultimately becomes undetectable [17,18]. The gradual decline of serum AMH concentration is parallel with the depletion of the number of growing ovarian follicles [20,21], which makes this hormone a reliable prognostic biomarker of ovarian reserve, as it is helpful in predicting the remaining length of the woman’s reproductive lifespan [22,23,24,25,26]. Moreover, since the serum AMH level reflects the quantity and quality of the ovarian follicular pool, it is also a dependable indicator of response to in vitro fertilization protocols in assessing the pregnancy success [17,27,28,29,30].

The persistence of AMH in males and females implies its multi-functional activity in both genders [31,32]. In the ovaries, AMH inhibits progesterone production, granulosa cell proliferation and primordial follicle activation [33,34,35,36]. In the testes, AMH influences androgen steroidogenesis by Leydig cells, whereas the stage-specific expression pattern within seminiferous tubules points to its substantial role in spermatogenesis [37,38]. Moreover, serum AMH is useful to monitor testicular function in boys and its low circulating levels may reflect primary testicular dysfunction, whereas undetectable levels indicate anorchidism or gonadal dysgenesis [39,40].

Clinical applications of AMH as a diagnostic biomarker and a promising therapeutic agent for AMH analogues have been developed recently [41,42]. Müllerian duct-derived tissues are the main source of various gynecologic tumors and since AMH causes regression of the male Müllerian ducts in male embryos by binding via tissue-specific AMHRII, it has also been proposed to inhibit the growth of gynecologic tumors [8]. In fact, AMH exerts an inhibitory effect by inducing apoptosis and cell cycle arrest in AMHRII positive endometrial cancer cell lines [8,43]. It has been assumed that the hormone may be a promising agent in effective treatment of various reproductive tract cancer types that express AMHRII, acting as an anti-cancer factor and cooperating with traditional chemotherapeutics [8,44,45,46]. High circulating AMH levels are supposed to be proportional to the protection effect against the development of endometrial cancer [47]. Furthermore, the serum AMH determination is useful to control the progress of ovarian granulosa cell and sex cord tumors [39,48,49]. Since the granulosa cell tumors secrete AMH in proportion to tumor burden, the changes in serum AMH reflect both recurrences and response to therapy [50,51,52].

As far, apart from testes and ovaries, AMH expression was confirmed in the endometrium of reproductive-age women, motoneurons of mice (where it works as protective factor) as well as in trace amounts in skeletal muscles, the sciatic nerve, the spinal cord and the brain of mice [53,54].

Endometrial cancer (EC) is the fourth most common cancer affecting women and the most common gynecologic cancer in developed countries [55,56]. Its established risk factors include obesity, use of exogenous estrogen after menopause, hypertension, diabetes, nulliparity, early menarche or late menopause [57,58]. Until now, data concerning the association between AMH and risks of endometrial cancer is still ambiguous [8,47,53]. Taking into consideration the above aspects, and the fact that AMH expression has not been previously investigated in EC, the goal of our study was to verify if there were associations between the AMH expression levels in tissues of various endometrial cancer types in terms of comorbidities, tumor malignancy, stage, histological type and grade.

## 2. Results

**Figure 1:**
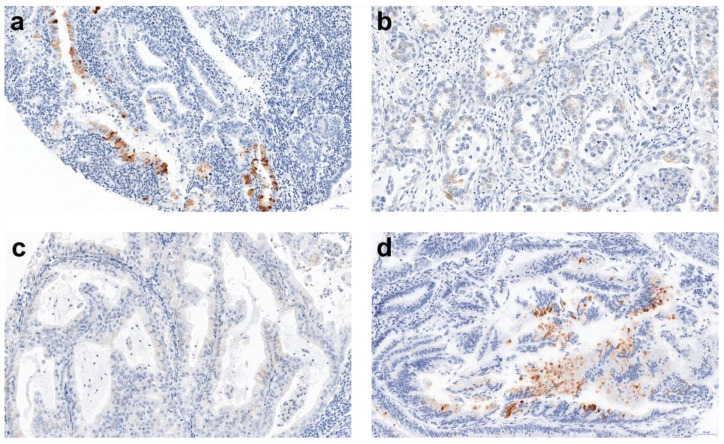
A representative example (20× magnification) of the immunohistochemistry (IHC) reaction: (**a**) well-differentiated (G1) endometrioid carcinoma showing focal but intense positive, membranous staining with AMH; (**b**) clear cell carcinoma showing few scattered anti-Müllerian hormone (AMH) positive cells with low intensity of staining. (**c**) AMH negative staining of well-differentiated (G1) endometrioid carcinoma; (**d**) well differentiated (G1) endometrioid carcinoma showing few scattered AMH positive cells with moderate intensity of staining.

All specimens were divided into eight groups, based on their histopathological type: endometrioid adenocarcinoma G1 (*n* = 49); G2 (*n* = 149); G3 (*n* = 6); nonatypical hyperplasia (*n* = 8), atypical hyperplasia (*n* = 4), serous adenocarcinoma (*n* = 8), clear cell adenocarcinoma (*n* = 5) and mixed adenocarcinoma (*n* = 5). Among 232 tissue microarray (TMA) specimens, 23 showed a positive AMH reaction (Figure 1). The detected AMH expression and its mean values are presented in Table 1.

For all patients, there were no differences in the overall AMH expression in the three collected tissue samples (F-test with Greenhouse-Geisser correction, ἐ = 0.714, *p* = 0.501), therefore in the analysis the average measure of AMH protein expression was used.

**Figure 2:**
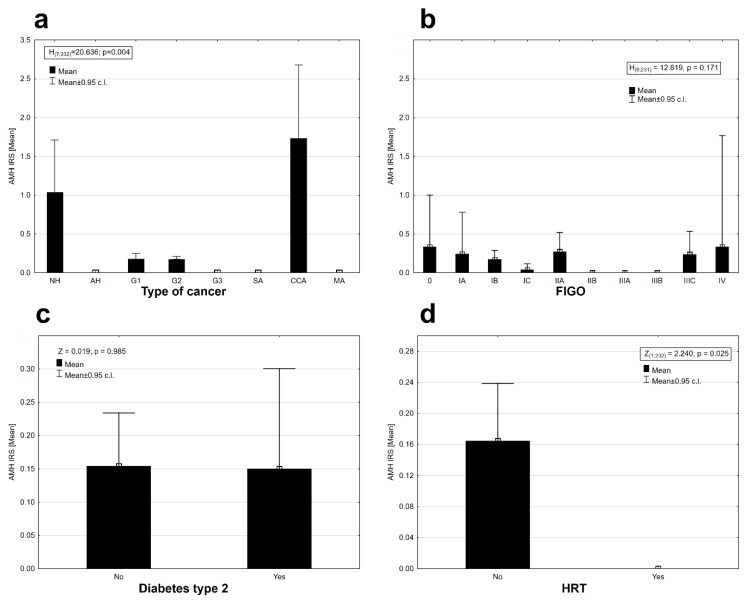
Mean AMH expression in: (**a**) different types of endometrial lesion (description of histopathological groups: NH—nonatypical hyperplasia, AH—atypical hyperplasia, G1—well differentiated endometroid adenocarcinoma, G2—moderately differentiated endometrioid adenocarcinoma, G3—poorly differentiated endometrioid adenocarcinoma, SA—serous adenocarcinoma, CCA—clear cell adenocarcioma, MA—mixed adenocarcinoma in Table 1) (Kruskal–Wallis ANOVA, H _(7, N = 232)_ = 20.636, *p* = 0.004); (**b**) in different clinical stages of endometrial cancer according to FIGO (International Federation of Gynecology and Obstetrics) 0—carcinoma in situ, IA —carcinoma limited to the inner lining of the uterus, IB—invasion less than half of the myometrium, IC—invasion equal to or more than half of the myometrium, IIA—invasion of the cervical glands, IIB —invasion of the cervical stroma, IIIA—involvement of the serosa or adnexa or both, IIIB—vaginal and/or parametrial involvement, IIIC—pelvic and/or paraaortic lymph node involvement IVA—Tumor invades bladder mucosa and/or bowel mucosa, IVB—Distant metastases (Kruskal–Wallis ANOVA, H _(9, N = 231)_ = 12.819, *p* = 0.171); (**c**) group of patients without and with diabetes mellitus type 2 (Mann–Whitney U test, AMH: Z = 0.019, *p* = 0.985); (**d**) group of patients that used hormone replacement therapy (Wald–Wolfowitz runs test, Z = 2.240, *p* = 0.025). IRS—immunoreactive score of Remmele and Stegner; c.l.—confident limits.

There was statistically significant differentiation of AMH protein expression between cancer types (Kruskal–Wallis ANOVA, H _(7, N = 232)_ = 20.636, *p* = 0.004). Expression was observed in the tissues of (pre-cancerous state) nonatypical hyperplasia, G1 and G2 cancers with a good prognosis, and in clear cell carcinomas (CCA) with a generally poor prognosis; the highest expression was observed in the clear cell carcinomas, slightly lower in the case of nonatypical hyperplasia and the lowest in good-prognosis G1 and G2 cancers (Figure 2a). No expression of AMH was observed in the case of other types of cancers (Figure 2a).

**Figure 3:**
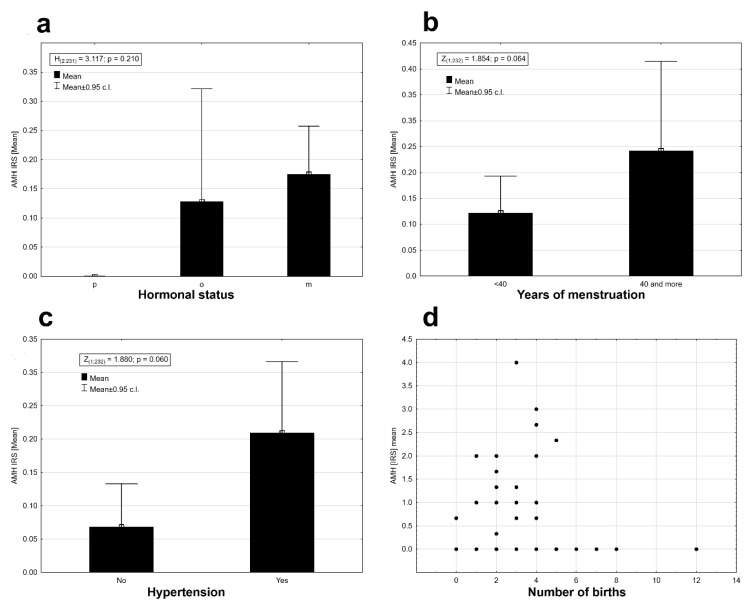
Mean AMH expression: (**a**) in premenopausal (p), perimenopausal (o) and postmenopausal (m) women (Kruskal–Wallis ANOVA: H _(2, N = 231)_ = 3.117, *p* = 0.210); (**b**) in the group of women that menstruated less than 40 years or more than 40 years (Mann–Whitney U test: Z = 1.854, *p* = 0.064); (**c**) in the group of patients without and with arterial hypertension (Mann–Whitney U test: Z = 1.880, *p* = 0.06); (**d**) depending on the number of births (r_s_ = 0.021, *n* = 232, *p* = 0.077).

**Figure 4:**
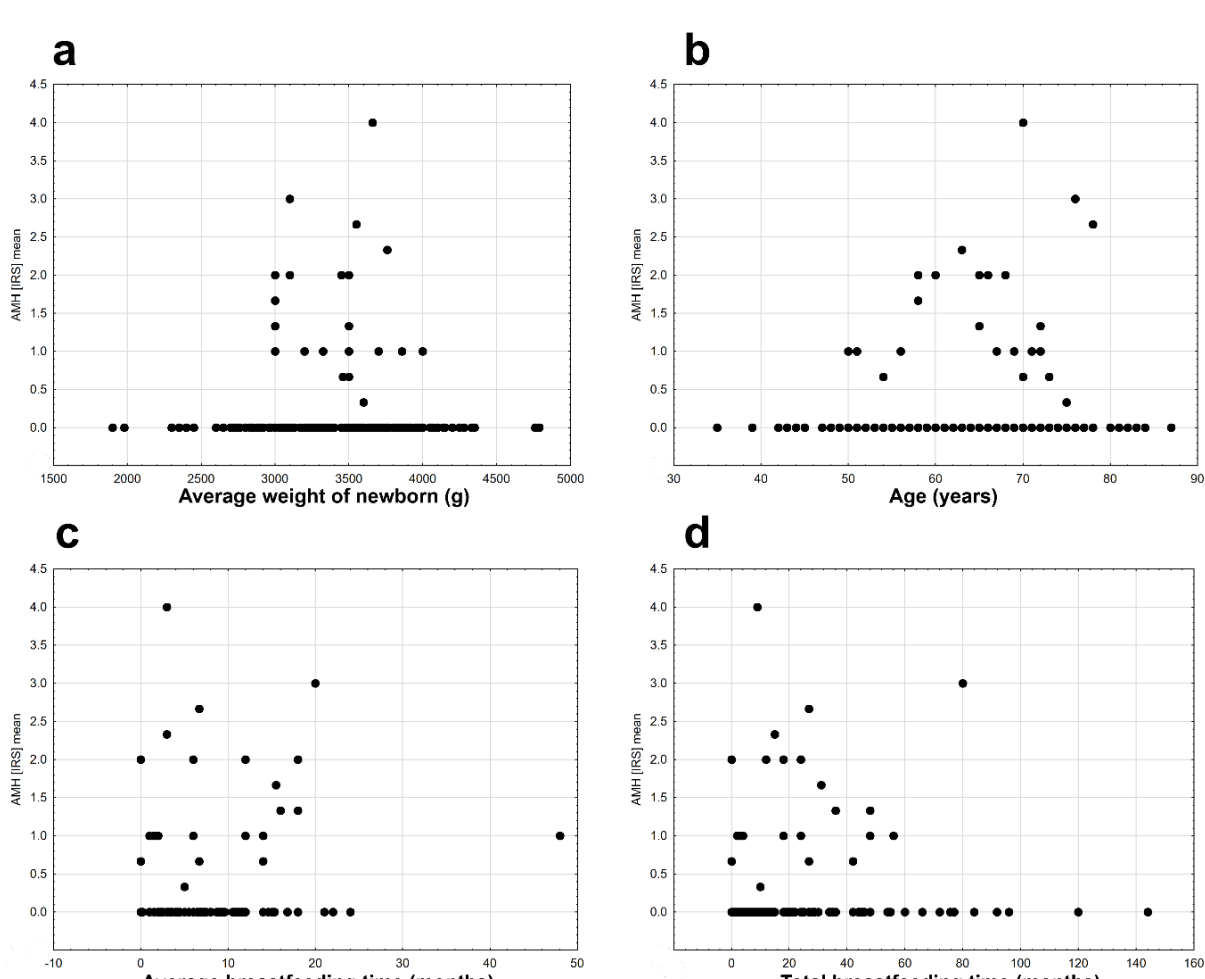
Mean AMH expression due to: (**a**) the weight of newborn (s) (r_s_ = −0.054, *n* = 207, *p* = 0.440); (**b**) age of the patient (r_s_ = −0.116, *n* = 232, *p* = 0.077); (**c**) mean breastfeeding time (r_s_ = −0.163, *n* = 229, *p* = 0.014); (**d**) total breastfeeding time (r_s_ = −0.134, *n* = 229, *p* = 0.042).

The AMH protein was found in some stages in the clinical staging system of cancer according to FIGO (International Federation of Gynecology and Obstetrics) staging (Figure 2b). The AMH protein was absent in the tissues of II B, IIIA, and IIIB FIGO stages. There was no statistically significant differentiation between the mean expression of the AMH protein in the stages of cancer according to FIGO (Kruskal–Wallis ANOVA, H _(9, N = 231)_ = 12.819, *p* = 0.171). Diabetes type 2 diagnosed before cancer did not affect the expression of AMH in EC tissues (Mann–Whitney U test, AMH: Z = 0.019, *p* = 0.985, Figure 2c). The presence of AMH protein was detected only in the group of patients who did not use hormonal replacement therapy (HRT; Figure 2d). Differences in AMH expression between both groups of women were statistically significant (Wald–Wolfowitz runs test, Z = 2.240, *p* = 0.025). AMH protein expression was observed in perimenopausal (o) and postmenopausal (m) women at similar levels (Figure 3a). Expression was absent in women who had premenopausal cancer (p) (Figure 3a). There were no statistical differences in AMH expression levels between the three groups of women (Kruskal–Wallis ANOVA: H _(2, N = 231)_ = 3.117, *p* = 0.210). Time from the first to the last menstrual bleeding of 40 or more years had an impact on the expression of AMH in EC tissues (Figure 3b), but the results were not statistically significant at the assumed level of I type error α = 0.05 (Mann–Whitney U test: Z = 1.854, *p* = 0.064). There were noticeable differences in the level of AMH protein expression depending on the presence or absence of arterial hypertension. Patients with arterial hypertension had a slightly higher expression of AMH (Figure 3c). The results were statistically insignificant at the assumed level of error α = 0.05 (Mann–Whitney U test: Z = 1.880, *p* = 0.06). There were no significant relationships between the AMH expression and the number of childbirths (deliveries) (r_s_ = 0.021, *n* = 232, *p* = 0.077, Figure 3d), the average birth weight of children (r_s_ = −0.054, *n* = 207, *p* = 0.440, Figure 4 a), and the age of the examined patients (r_s_ = −0.116, *n* = 232, *p* = 0.077, Figure 4b). Significant but very weak positive relationships were found between AMH expression and mean breastfeeding time (r_s_ = −0.163, *n* = 229, *p* = 0.014, Figure 4c) and total breastfeeding time (r_s_ = −0.134, *n* = 229, *p* = 0.042, Figure 4d).

It was found that the probability of AMH protein expression was significant depending on the average breastfeeding time (*p* = 0.004), the type of cancer (*p* = 0.006) and menstrual years (*p* = 0.045, Table 2). No significant interactions between the variables studied were found. The probability of AMH protein expression elevates with the increase in the average breastfeeding time, type of cancer (significantly increases the expression of type G2, G1 and CCA tumors) and depending on the years of menstruation (women with menstruation below 40 years had less expression of AMH, Table 3).

## 3. Discussion

The normal levels of serum AMH in women between puberty and menopause amount to 1.4–5 ng/mL [15,59], and then it decreases to undetectable values [60]. The highest reported serum AMH concentration of 3205.93 ng/mL was found in a patient with sex cord tumor in whom remote metastases were present [50]. Sex cord tumors are rare and may also be analyzed in the context of Peutz–Jeghers syndrome [61]. Determination of serum AMH is used in diagnosing granulosa cell tumors [52]. A positive correlation was found between the AMH level and gross aggregate tumor mass determined by pathology, as well as between the AMH level and radiographic aggregate tumor mass [52]. In patients with this type of cancer, the serum AMH level reached 1200 ng/mL [52]. Serial measurement of serum AMH in granulosa cell tumor patients is performed in order to assess the efficacy of surgical treatment and to monitor possible relapses of the disease [62]. Elevated levels of AMH are observed also in patients with polycystic ovary syndrome (PCOS), where they are elevated up to 2–12 times [63,64]. This is connected to a higher number of small follicles in a group of these women [65]. Determination of serum AMH levels, apart from being a reliable assessment of the ovarian reserve, helps to individualize the dosing of follitropin alfa (rFSH) in artificial reproductive techniques (ART), which helps to reduce side effects of ovarian hyperstimulation [28,29,66].

AMH expression was found in mitosing cells of the endometrium of reproductive-age women [53]. Its expression increases in presence of both sex steroid hormones—progesterone and estradiol [53]. AMH derived from endometrium has the potential to elicit apoptosis and decrease viability of endometrial cells [53]. Serum AMH present in referential concentrations until menopause may exert protective action on female organisms and inhibit the development of EC. This was confirmed by the average age of disease onset at 62.5 years in the group of women analyzed in this study. The most important number of cases of this type of cancer was observed in postmenopausal women (80.49%), when AMH levels dropped to undetectable values. In our series, AMH protein was rarely found in the analyzed EC samples. Although the age alone did not have a significant impact on AMH expression in EC cells, our study confirmed that AMH protein was absent in women who developed EC at the premenopausal age (0/24). Another author did not find a correlation between serum AMH levels and diagnosing EC at the premenopausal age [47]. Possibly, AMH derived from both tissues—ovary and endometrium—may negatively influence EC development while working together. In analysis of EC etiology one should also consider other factors including obesity, hypertension and diabetes, as their correlation with EC has already been proven [67,68,69]. Although hypertension was related to elevated AMH expression in EC cells (statistically not significant), neither type 2-diabetes, nor BMI correlated with AMH expression. Due to increased levels of AMH in PCOS [63,64,70], a correlation between EC and PCOS is questionable [70]. This doubt is supported by epidemiological data—annually, some 4000 new cases of EC are diagnosed in the UK, while the number of PCOS patients in this country is estimated to be 500,000 to 1 million [70].

The widely known negative relationship between parity and EC indicates that multiparity is a factor protecting women against EC. In our study the presence of the AMH protein in EC cells did not correlate with the number of child births and birth weight of newborns. The AMH protein was detected only in the group of patients who did not use HRT. However, as the patients’ history revealed, only 15 patients used HRT for more than 6 months. Yet, a correlation was observed between elevated AMH expression and the length of life hormonal activity, that is the time from the first to the last menstrual period. This phenomenon was not observed when the time of estrogen activity was shorter than 40 years. Among women who menstruated 40 years and more, 82.92% were patients with diagnosed G1 and G2 endometrioid carcinomas. In conjunction with AMH expression, this coincides with a good prognosis in the hormone-dependent type of cancer. Similarly, a longer average period of breastfeeding in conjunction with G1, G2 or CCA histopathological type is a factor increasing AMH expression. We found no differences between the stages of cancer according to FIGO and AMH expression. Although the protein was not detected in IIB, IIIA and IIIB stages, it was present in IIIC and IV stages. This observation seems to correlate with the determined elevated levels AMH concentration in cases of cancer which spread outside the uterus, in contrast to low levels of AMH in patients with cancer limited only to the uterus [71].

In analysis of the histopathological type of cancer, the probability of detecting AMH in cells increased in cases of nonatypical hyperplasia, as well as G1 and G2 endometrioid type of EC and, surprisingly in CCA. Patients with the CCA and positive AMH expression demonstrated IA-IB clinical stage of disease. None of them were obese (BMI 17.1–25.9) and their average age was 71.6 years. This justifies undertaking further research on AMH expression and a 5-year survival period in patients with clear cell carcinomas. The presence of AMH in type II cancers according to Bokhman’s taxonomy might be the reason behind their biological diversity and a better than average survival rate in this type of cancer.

AMH is a natural substance which induces cell cycle arrest and apoptosis, with its activity limited to a few tissues. Thus, it was conceived that AMH represents a non-toxic substance which may be potentially useful in treating cancers exhibiting AMH receptors [8,16,72,73,74].

The efficacy of controlling the development of mouse ovarian carcinoma (MOVCAR) cells was confirmed with recombinant human AMH, with no symptoms of toxicity during a 11-week treatment, equivalent to a continuous 7-year treatment in humans [75].

The neoplastic process in the endometrium engages some 1000 genes—362 up-regulated and 638 down-regulated ones [76]. It was shown that applying AMH in EC tissue changes the activity of 2688 genes engaged in regulating the cell cycle and apoptosis [77]. Expression grows in, among others, apoptotic protease activating factor-1 (*APAF-1*), β-catenin-interacting protein (*ICAT*), Rb related protein 130 (p130), while it decreases in, among others, cyclin-dependent kinase 2 (*CDK2*) and phospho-c-Jun [77]. Understanding the mechanisms leading to proapoptotic and cell cycle arrest functions of AMH is of key importance in order to use this substance as a therapeutic protein agent.

AMH uses various signaling pathways and inhibits cell divisions or programmed cell death in particular tissues in distinct ways. In the tissue of endometrial cysts of the ovary, AMH increases the concentration of p53-dependent p21 protein (cyclin-dependent kinase inhibitor—CDK inhibitor), as well as p107 and p130 proteins from Retinoblastoma family, while it decreases the level of transcription factor E2F1 [78]. AMH exhibits a similar activity in EC [43]. In the ovarian carcinoma, it increases the level of CDKs inhibitors: p16 and p21 [79]. Due to the activity of AMH, the levels of p16, p107 and p130 increase in cervical carcinoma cells [80].

The new classification of EC according to The Cancer Genome Atlas Research Network (TCGA) distinguishes four EC groups [81,82]. Group 4, labelled “copy-number high (serous-like),” encompasses cancers with the most serious prognosis [81,82]. Analyzing mutations from which the neoplastic process derives, it may be concluded that about 92% of cancers in this group present mutations of the p53 gene [81,82]. Theoretically, AMH—as an adjuvant—could increase the efficacy of treatment in the worst types of EC mediating the increased levels of p21.

In breast cancer and prostate cancer, AMH activates the pathway of NFκB (nuclear factor kappa-light-chain-enhancer of activated B cells), which induces the *IEX* gene (immediate early gene), encoding the protein regulator of the cell cycle [83,84,85]. It was demonstrated that in the T47D estrogen-positive line of breast cancer cells AMH causes selective expression of mRNA *IEX-1S* splice variant, and *IEX-1L* variant, which is responsible for the survival of colonies of cells, was absent [45,86]. AMH causes a similar response in the estrogen-negative line of breast cancer (MDA-MB-231) [38]. Transcripts of both *IEX* variants were determined, but only *IEX-1S* reached biologically significant levels, which resulted in 50% cell cycle arrest [45].

AMH sensitizes malignant ovarian cells to chemotherapy, increasing its efficacy [46,87,88]. Current precise onco-therapy encompasses blocking receptor tyrosine kinases (RTKs) [89,90]. The development of resistance in EC cells to modern medication relies on downregulation of *PHLDA1* (pleckstrin homology-like domain family A member 1), a protein regulating apoptosis [91,92]. *PHLDA1* expression is responsible for basal apoptosis, impedes the growth of neoplastic cells and sensitizes the neoplastic tissue to chemotherapeutics [93,94]. Activation of the NFκB pathway leads to upregulation of *PHLDA1* [91]. It is not known whether the activation of this pathway by AMH has any effect on the level of *PHLDA1*. If it does, AMH could support the activity of RTKs antibodies and counteract the development of resistance towards them.

It was found that single nucleotides polymorphism (SNP) can be responsible for the development of cancers, including EC [95,96,97,98,99]. Overexpression of the murine double minute 2 gene (*MDM2*) leads to inhibiting the activity of p53 protein, which, in consequence, causes an increased risk of cancer [100,101,102]. Nucleotide 309 polymorphism (SNP309) in the first intron of the *MDM2* gene (rs2279744) is a risk factor for EC among Caucasian and Asian women, as a result of an increased level of *MDM2* [103,104]. Worth considering is whether applying AMH in the adjuvant EC therapy in cases of SNPT309G, in the presence of AMHERII, may increase the efficacy of treatment in this type of cancer.

Belonging to the TGFβ superfamily, AMH acts through signaling pathways related to the SMAD protein and engages slightly different mechanisms and transmitter proteins than the constitutive proteins of TGFβ [105,106,107,108]. These proteins activate a non-canonical signaling pathway which leads to activating proapoptotic p38/mitogen-activated protein kinase (p38/MAPK) [109,110]. The TGFβ superfamily does not show, however, an antiproliferative effect in the case of constitutive overexpression or transient overexpression of *MDM2* [111,112]. It is not known whether AMH, using other pathways than the classic TGFβ proteins [87,113], is also lacking this function.

It is believed that epigenetic modulations have a growing role in the neoplastic process. There are two opposed systems which maintain or suppress the activity of genes through remodeling of chromatin: Polycomb (PcG) and Trithorax (TrxG) [114,115,116]. An important component of Polycomb which is responsible for the development of EC is an enhancer of zeste homolog 2 (*EZH2*), which impedes the activity of suppressor genes [117,118]. Knockdown of the *EZH2* gene leads to apoptosis of EC cells because of an increase in the level of caspase-3 and caspase-9 [117]. AMH also increases the level of caspase-3 [87]. In such cases AMH could also find a therapeutic application.

Among natural substances, not only AMH has a beneficial effect on the reduction of EC cells. Hesperidin (a flavonoid from Citrus species) induces apoptosis through p38/mitogen-activated protein kinase [119]. Eupatilin (from *Artemisia princemps*) increases the level of the p21 protein and inhibits the growth of EC cells in the G2/M stage [120].

Summarizing, as the knowledge on carcinogenesis and its molecular basis increases, novel or modified therapeutic solutions appear in modern oncology. A member of the TGFβ family, AMH represents a substance which should be focused on in 21st century medicine because of its unique properties and safety profile.

## 4. Materials and Methods

### 4.1. Ethics Statement and Research Material

The experimental material was collected at the Clinical Ward of Gynecology, Obstetrics and Oncological Gynecology at the Regional Specialist Hospital in Olsztyn, Poland. The study protocol was approved by the Bioethics Committee of the Warmia-Mazury Medical Chamber (OIL.164/15/Bioet; 2 April 2015) in Olsztyn, Poland. Case history reviews were collected for all patients in order to record demographic details and their whole medical history. The classification of patients according to confirmed postoperative histopathological type is presented in Table 4.

For each patient medical data such as, the type of cancer, cancer stages according to FIGO staging, hormonal status of women, menstrual activity, presence of type 2 diabetes, hypertension, use of HRT, number of births, time of breastfeeding, age of women and body mass index (BMI), were collected. According to the World Health Organization (https://www.who.int/topics/obesity/en/) the BMI values correspond to underweight (BMI < 18.5 kg/m^2^), normal weight (BMI = 18.5–25), overweight (BMI = 25–30), and obese (BMI > 30) [121]. According to the British guidelines (National Collaborating Centre for Women’s and Children’s Health, 2015) the perimenopausal period is the time of irregular menstruations and vasomotor symptoms [122]. The menopausal period means that a woman has not had menstruation for at least 12 months and she does not use hormonal contraception [122].

The biopsy specimens were obtained from 232 patients during surgical interventions consisting of hysterectomy with bilateral salpingo-oophorectomy, lymphadenectomy (except pre-cancerous states of endometrium (PCS) cases) and peritoneal washing. The surgical biopsies from the affected area were preserved in 4% buffered formalin/formaldehyde immediately after surgical removal in the form of a phosphate-buffered solution (Chempur, Piekary Śląskie, Poland). The volume of fixative to tissue ratio was at least 10:1. The specimens were fixed by immersion for 6 to 12 h before further processing. The preserved tissues were placed in cassettes and the batches of specimens were loaded onto a tissue processor (Leica ASP 300S, Leica Biosystems, Nussloch, Germany) before being molten in wax. Embedding was performed 10 h later on the platform (Leica EG1160). Paraffin blocks were cut Leica SM 2000R microtome into 6 μm-thick sections. Following in time flattening and straightening on the surface of warm water (37 °C) they were picked up onto microscope slides. After thorough drying (overnight/37 °C) specimens were stained in the hematoxylin and eosin (H&E) automated stainer (Leica ST5020) for visualization of particular structures. After staining, the sections were covered with a glass coverslip and evaluated by a qualified pathologist. The Formalin-Fixed Paraffin-Embedded (FFPE) specimens were processed according to the College of American Pathologists (CAP) criteria described in The Practical Guide to Specimen Handling in Surgical Pathology [123].

### 4.2. Tissue Microarrays (TMAs)

The prepared slides were scanned using the Panoramic MIDI II (3DHistech, Budapest, Hungary) histological scanner. Panoramic Viewer (3DHistech) software was used to select manually three representative areas (each with a surface of 1.5 mm^2^) from regions of EC previously indicated by the pathologist. Three representative cores of 1.5 diameter mm were taken from each archival tumor sample of an EC and embedded in paraffin as described above to create tissue microarrays (TMAs) using TMA Grand Master (3DHistech, Budapest, Hungary) in line with the manufacturer’s instructions.

### 4.3. Immunohistochemistry (IHC)

Slides from TMAs (4 μm-thick) were used for immunohistochemistry (IHC) reactions, which were performed using DakoAutostainer Link48 (Dako, Glostrup, Denmark). In order to deparaffinize, rehydrate and unmask the antigens the sections were boiled in EnVision FLEX Target Retrieval Solution (pH 9, 20 min, 97 °C; Dako) using the PTLink platform (Dako, Glostrup, Denmark). Afterwards, slides were incubated for 5 min with Envision Flex Peroxidase-Blocking Reagent (Dako, Glostrup, Denmark) to block endogenous peroxidase. As primary antibodies (20 min, RT), rabbit polyclonal antibodies against AMH (1:100, ab84952, Abcam, Cambridge, UK) were used. Next, slides were incubated with EnVision FLEX/HRP (20 min, RT), and the reaction was visualized (10 min, RT) with freshly prepared 3,3′-diaminobenzidine (DAB). Additionally, slides were counterstained for 5 min with EnVision FLEX Hematoxylin (Dako, Glostrup, Denmark). Finally, slides were dehydrated in ethanol (70%, 96%, absolute) and xylene, then mounted with Dako Mounting Medium (Dako, Glostrup, Denmark). Slides were evaluated using the Olympus BX41 light microscope (Olympus, Japan). Control tissues included the human prostate.

### 4.4. Evaluation of IHC Reactions

All immunohistochemical reactions were evaluated by two pathologists using a BX-41 light microscope. For evaluation an immunoreactive score of Remmele and Stegner was applied [124] (Table 5).

### 4.5. Statistical Analysis

The expression of AMH was measured with the rank scale IRS immunoreactive score (IRS) of Remmele and Stegner (Table 5) in three samples retrieved from three sites of cancer tissue from each patient. There were no differences in the overall AMH expression in the three collected tissue samples (F-test with Greenhouse-Geisser correction, ἐ = 0.714, *p* = 0.501). The mean value of AMH expression for each patient was computed, and the mean values were used in the whole analysis. Differences in AMH expression between the type of cancer, cancer stages according to FIGO, hormonal status of women were tested with the Kruskal–Wallis test. Results of comparisons between individual groups were based on the post hoc nonparametric multiple comparison tests. The comparison of AMH expression in two groups of patients, e.g., years of menstrual activity (<40 years old, 40 or more years old), presence of type 2 diabetes, presence of hypertension, the use of hormonal replacement therapy (HRT), was performed with the Mann–Whitney U test or the Wald–Wolfowitz runs test. The relationship between AMH expression and values of metric traits (number of births, time of breastfeeding, BMI, age of women) was tested using Spearman’s rank correlation coefficient. The multidimensional comparison of AMH expression (binary data: expression positive—1, and negative —0) and an independent category and continuous variables used in the study were tested in Generalized Linear Model (GLZ) with logit linking function and searching for the best fitted models with the Akaike criterion. The parameters were estimated using the highest likelihood method. In the case of variable redundancy, the parameter was not estimated. The *p*-value < 0.05 was defined as statistically significant. Statistical analysis was performed using Statistica 13.0 software (TIBCO Software Inc. 2017. Statistica (data analysis software system), version 13. http://statistica.io, Krakow, Poland)).

## Figures and Tables

**Table 1:** Summary of histopathological type of endometrial lesion, number of patients in each group and mean, minimal and maximal values of AMH expression (F-test with Greenhouse-Geisser correction, ἐ = 0.714, *p* = 0.501).

Histopathological Type of Endometrial Lesion	Number of Patients in Each Group	Number of Patients with Positive AMH Expression	Mean Value of AMH Expression	Minimal AMH Expression	Maximal AMH Expression	SD
Nonatypical endometrial hyperplasia (NH)	8	2	2.17	0.33	4.0	2.593
Atypical hyperplasia (AH)	4	0	-	-	-	-
Endometroid adenocarcinoma G1 (G1)	49	3	1.78	1.0	3.0	1.072
Endometroid adenocarcinoma G2 (G2)	147	15	1.33	0.67	2.33	0.590
Endometroid adenocarcinoma G3 (G3)	6	0	-	-	-	-
Serous adenocarcinoma (SA)	8	0	-	-	-	-
Clear cell adenocarcinoma (CCA)	5	3	2.0	1.33	2.67	0.667
Mixed adenocarcinoma (MA)	5	0	-	-	-	-

**Table 2:** Model of relations between the AMH protein expression (modeled probability = 1) and studied variables; the best flitted model using the AIC (Akaike information criterion) (AIC = −128.102; df = 9; χ2 = 27.795; *p* = 0.001).

Variable	df	Max. Likelihood	χ2	*p*
Average breastfeeding time (continuous variable)	1	−58.182	8.262	0.004
Cancer type (NH, G1, G2, G3, SA, CCA, MA)	6	−63.122	18.142	0.006
Menstrual year (0—below 40; 1—40 and more)	1	−56.062	4.022	0.045

**Table 3:** Parameters of the best fitted model of modelled probability of AMH protein expression and studied variables; model reduced to the all significant levels of independent variables. c.l.—confident limits. a—intercept (point where the regression line crosses the axis).

Variable	Level of Variable	Parameter ± SE	Lower 95.0% c.l.	Upper 95.0% c.l.	Wald Function	*p*
a		−11.274 ± 0.859	−9.590	−12.958	172.174	0.000
Average breastfeeding time	continuous	0.091 ± 0.032	0.154	0.028	7.936	0.005
Type of cancer	G2	8.417 ± 0.925	10.230	6.604	82.794	0.000
	G1	8.125 ± 1.051	10.186	6.065	59.744	0.000
	CCA	12.038 ± 1.445	14.870	9.205	69.362	0.000
Menstrual year	0—below 40	−0.555 ± 0.273	−0.019	−1.090	4.125	0.042

**Table 4:** Number of patients with different histopathological types of endometrial lesions.

Histopathological Type of Endometrial lesion	Number of Patients
Nonatypical endometrial hyperplasia	8
Atypical hyperplasia	4
Endometrioid adenocarcinoma G1	49
Endometrioid adenocarcinoma G2	147
Endometrioid adenocarcinoma G3	6
Serous adenocarcinoma	8
Clear cell adenocarcinoma	5
Mixed adenocarcinoma	5

**Table 5:** Remmele and Stegner score: the percentage of positive cells (A) and the intensity of color reaction (B). The final score represents the result of these parameters (A × B)/semi-quantitative immunoreactive score (IRS) of Remmele and Stegner. pt—point, pts—points.

A	B
0 pts—no cells with positive reaction	0 pts—no staining
1 pt—<10% cells with positive reaction	1 pt—low intensity of staining
2 pts—11%–50% cells with positive reaction	2 pts—moderate intensity of staining
3 pts—51%–80% cells with positive reaction	3 pts—intense staining
4 pts—>80% cells with positive reaction	

## Abbreviations

MDPI	Multidisciplinary Digital Publishing Institute
DOAJ	Directory of open access journals
TLA	Three letter acronym
LD	linear dichroism
